# A multiscale chemical-mechanical model predicts impact of morphogen spreading on tissue growth

**DOI:** 10.1038/s41540-023-00278-5

**Published:** 2023-05-20

**Authors:** Alireza Ramezani, Samuel Britton, Roya Zandi, Mark Alber, Ali Nematbakhsh, Weitao Chen

**Affiliations:** 1 Department of Physics and Astronomy, University of California, Riverside, CA 92521 USA; 2 Interdisciplinary Center for Quantitative Modeling in Biology, University of California, Riverside, CA 92521 USA; 3 Department of Mathematics, University of California, Riverside, CA 92521 USA

**Keywords:** Computational science, Software

## Abstract

The exact mechanism controlling cell growth remains a grand challenge in developmental biology and regenerative medicine. The *Drosophila* wing disc tissue serves as an ideal biological model to study mechanisms involved in growth regulation. Most existing computational models for studying tissue growth focus specifically on either chemical signals or mechanical forces. Here we developed a multiscale chemical-mechanical model to investigate the growth regulation mechanism based on the dynamics of a morphogen gradient. By comparing the spatial distribution of dividing cells and the overall tissue shape obtained in model simulations with experimental data of the wing disc, it is shown that the size of the domain of the Dpp morphogen is critical in determining tissue size and shape. A larger tissue size with a faster growth rate and more symmetric shape can be achieved if the Dpp gradient spreads in a larger domain. Together with Dpp absorbance at the peripheral zone, the feedback regulation that downregulates Dpp receptors on the cell membrane allows for further spreading of the morphogen away from its source region, resulting in prolonged tissue growth at a more spatially homogeneous growth rate.

## Introduction

Understanding mechanisms underlying proper tissue growth and shape formation in an embryo is among the most important unanswered questions in developmental biology. The growth of tissues and organs always exhibits the property of self-organization, with precise control of cell proliferation resulting in robust tissue size and specific shape as integrity. This process also stays robust under external perturbations as observed in wound healing and tissue regeneration^1–6^. Uncontrolled cell growth and cell division often lead to abnormal development or fatal diseases such as cancer.

During tissue development, chemical signals are found to be critical to the regulation of cell proliferation and tissue shape formation. A variety of molecules, from extracellular ligands to intracellular proteins, have been identified as growth regulators in different biological systems. For example, transforming growth factor Beta (TGF-β), a member of the growth factor superfamily, has been found to regulate the growth in multiple animal organs^7,8^. In particular, bone morphogenic proteins (BMP) are members of the TGF-β family and play essential roles in establishing the basic embryonic body plan for tissue development in vertebrates^9–13^. Disruption of BMP signals can affect the growth rate and pattern formation, leading to disorders in adult tissues^14,15^. On the other hand, in addition to the central core of the growth control machinery, which depends on chemical cues, cell mechanics play a fundamental role in shaping a tissue^16–23^. Each cell has a complex mechanical architecture that not only shapes itself as integrity but also allows it to sense the physical surroundings and make responses. For example, cytoskeletal tension in one cell can be affected by differential growth associated with neighboring cells and modulate intracellular molecular signals to regulate growth as feedback^24^. Cell deformation can be induced by mechanical forces such as the adhesion to the extracellular matrix (ECM), contractility in the cytoskeleton, and cell–cell adhesion, which may also lead to physical changes of nuclei and an alteration in gene expression to switch cell fate between growth, differentiation, and apoptosis^25^. Therefore, it is necessary to consider both chemical signals and mechanical properties, as well as the interplay between them, to understand the general principles involved in tissue development.

**Fig. 1:**
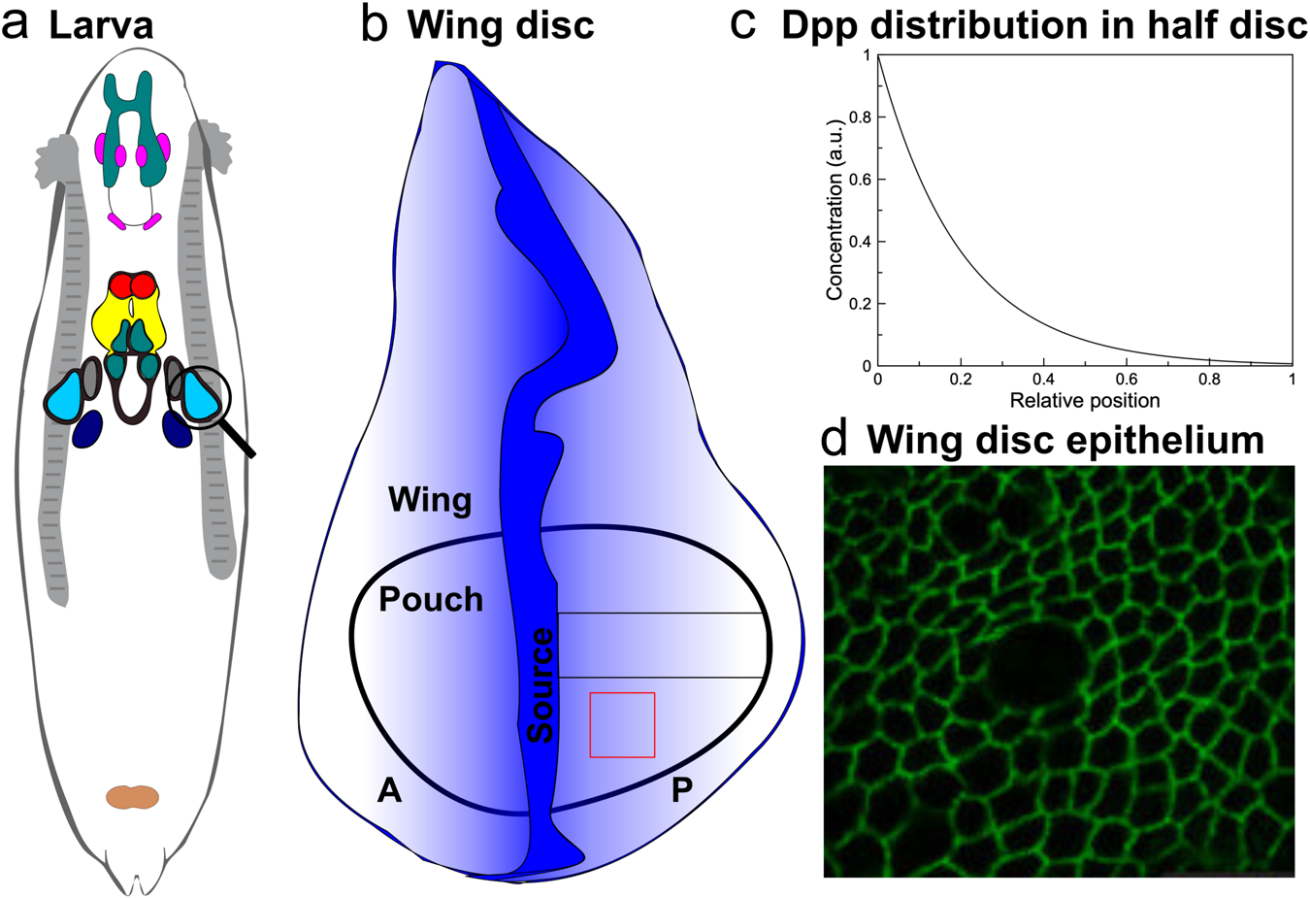
Overview of wing disc epithelium and morphogen gradient. **a** Diagram of *Drosophila* larva with wing disc tissue circled. **b** Illustrative diagram of the *Drosophila* imaginal wing disc. The blue color denotes the Dpp morphogen gradient. **c** Schematic profile of the Dpp morphogen in half wing disc. Its distribution follows an exponential shape, as observed in experiments. **d** Configuration of epithelial cells in the wing disc pouch. The image has been reproduced from Gibson et al.^26^.

*Drosophila* wing disc, a primordial epithelial organ that later becomes the adult wing, as shown in Fig. 1a, serves as a classic model to study tissue growth regulation due to its simple geometry, the limited number of cells, and fast growth. Additionally, the well-established molecular signaling network involved in this tissue contains multiple conserved molecules critical to other developing systems in mammals^8^. Understanding the mechanism of growth regulation in the *Drosophila* wing disc is substantial in understanding limb development in mammals. In this tissue, Decapentaplegic (Dpp), a homolog of BMP, forms a spatial gradient across the anterior-posterior (AP) axis of the tissue to establish and maintain domains of multiple target genes that specify different compartments in the adult tissue (Fig. 1b, c). For individual cells, a signal transduction cascade converts local concentrations of Dpp into intracellular phosphorylated MAD (pMAD) through binding with receptors on the membrane. pMAD protein is also commonly observed in other systems and related to several cancers in humans^7^. Based on the level of pMAD, different genes are activated along the AP axis of the imaginal wing disc to establish the pattern and regulate growth. In terms of mechanical properties, a wing disc consists of a flat sheet of cells with E-cadherin responsible for cell–cell adhesion between neighboring cells. Inside individual cells, actomyosin is dynamically rearranged to give rise to different levels of contractility, which links to multiple cellular functions, including nuclear motion during mitotic rounding (Fig. 1d)^26^ and vesicle trafficking^27,28^. Moreover, actin networks in the cytoplasm, as a major component of the cytoskeleton, provide structural support to each cell and determine cell shapes together with the cytoplasm. More recently, it has been observed that chemical signals can affect cell mechanics by directly controlling the subcellular distribution of the small GTPase Rho1 and the regulatory light chain of non-muscle myosin^29^. Dpp signal promotes the compartmentalization of Rho1 and myosin, which leads to the contraction of actomyosin filaments and an increase in cortical tension. This suggests the interaction between chemical signals and mechanical properties also plays an important role in shaping cells and, therefore, the overall tissue shape.

Several hypotheses regarding growth regulation in the wing disc tissue have been proposed so far. Substantial data suggest that Dpp morphogen is pivotal in regulating growth; however, the underlying mechanism remains controversial and uncertain. In Wartlick et al.^30^, it was suggested that cells have memory and will divide if the temporal change of the Dpp signal reaches a certain threshold value. In contrast, recent experiments have shown that a Dpp signal is not always required for growth since removing Dpp from the center of the tissue at some stage during the development does not affect the growth^31^. On the other hand, mechanical properties have been shown to be critical in regulating growth based on measurements of cell stress in experiments^24^.

Many computational models have been developed to study tissue growth in different biological systems, including cell lineage in epithelia^32–35^ and tumor growth^36–38^. To include chemical signaling networks, it is common to use continuous models in which the dynamics of chemical signals are captured by a system of differential equations. This kind of approach usually involves moving boundary problems for capturing tissue growth that are challenging to solve numerically. It can be overcome by using Lagrangian coordinates^39^, immersed boundary method^40^, level set method^41^, or other similar approaches. To include cell mechanics, some models use discrete particles to represent individual cells, which allows one to model cell growth, cell division, and cell–cell interaction. More specifically, each cell can be represented by a single particle (agent-based model)^42^, multiple particles on the cell membrane (multi-agent-based model)^43^, a polygon (vertex-based model)^21,30,44,45^, or multiple particles on the cell membrane and in the cytoplasm (subcellular element method)^46^. In particular, multi-agent-based models and subcellular element models can describe biologically relevant cell shapes with greater flexibility due to the multiple nodes involved. Another type of model is based on the finite element framework, coupled with continuum mechanics principles^47–50^. Models of this type focus more on tissue growth without subcellular details. Most existing models for studying tissue growth focus on either chemical signals or mechanical properties only. As suggested by recent experimental data, exploring mechanisms involved in tissue growth regulation requires a model that includes both chemical and mechanical factors, as well as the interactions between them.

Several coupled chemical-mechanical models have been developed recently and gained lots of attention. In Aegerter-Wilmsen et al.^51^, both chemical signals and mechanical cues were considered. However, fixed morphogen gradients were adopted without considering the temporal dynamics or subcellular activities. Vertex-based models have been coupled at the cell level with diffusive molecules^52^ or intracellular gene expression^7,8^ to study tissue development. The subcellular element model has also been coupled with chemical signals without distinguishing cell membrane and cytoplasm^53,54^. Those existing models provide novel insights into growth regulation in different systems. As far as we know, very few of them consider subcellular details and the interaction between chemical signals and mechanical forces, which is critical in the regulation of individual cell behavior and tissue growth.

In this study, we developed a multiscale coupled chemical-mechanical model where the mechanical submodel describes cell mechanical and adhesive properties at the subcellular and cellular levels, and the chemical signaling submodel describes both morphogen gradient at the tissue level and the intracellular gene regulatory network at the cellular level. This model was then applied to study growth regulation in *Drosophila* wing imaginal disc. In addition, we incorporated a cell division rule proposed in Wartlick et al.^30^, in which cells enter the mitotic phase and divide when the Dpp signal is increased by 50% compared with that at the beginning of the cell cycle in individual cells. Following this hypothesis and including cell mechanical properties, morphogen gradients with different decay lengths were tested in the model to simulate tissue growth. We found that a morphogen gradient with a larger decay length maintained the tissue growth longer, resulting in a more symmetric shape at a more spatially homogeneous growth rate, which was consistent with experimental observations. Together with the assumption of an absorbing boundary condition, feedback regulation of the downstream signal to inhibit the synthesis of cell membrane receptors facilitated tissue growth by indirectly expanding the spread of the morphogen gradient. Although the chemical-mechanical model was only applied to studying the growth regulation of *Drosophila* wing disc, it can be applied to simulate tissue growth and test hypotheses on growth regulation involved in other epithelial tissues.

## Results

We developed a two-dimensional chemical-mechanical model for studying tissue development and applied it to explore the growth regulation mechanism in the *Drosophila* wing disc. In particular, we aimed to understand how spatially uniform growth can be achieved and maintained throughout tissue development, as observed in experiments.

### Multiscale chemical-mechanical model of tissue development in two dimensions

**Fig. 2:**
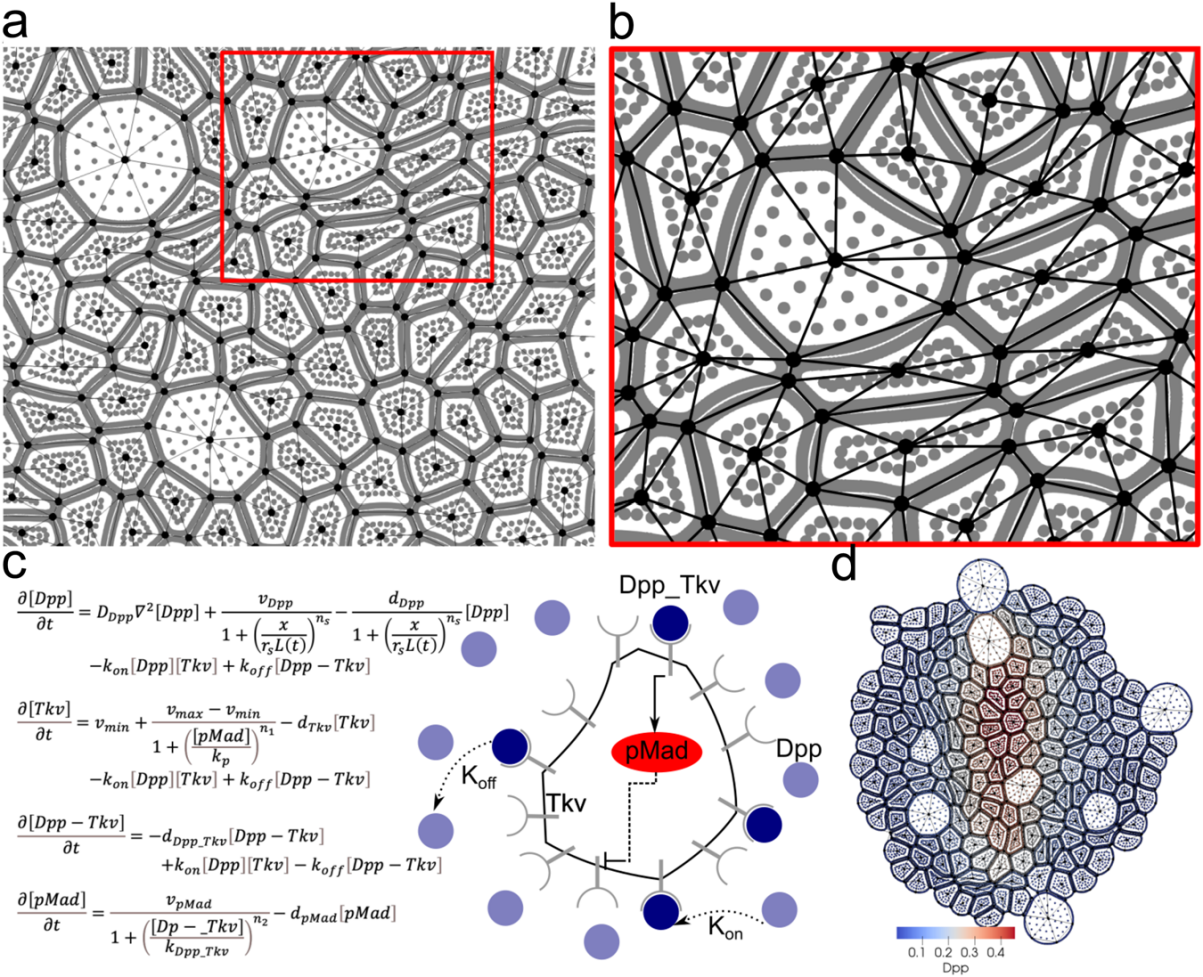
Spatial coupling of chemical and mechanical submodels. **a** Triangular mesh over the nodes obtained in the mechanical submodel. **b** A zoom-in view of the triangular mesh within the red box indicated in (**a**). **c** Mathematical model (left) and a schematic diagram (right) of the chemical signaling network in a single cell of *Drosophila* wing disc. **d** Discretized tissue with Dpp gradient, denoted by blue-red color, obtained in the chemical-mechanical model.

During tissue development, both chemical signals and mechanical forces play essential roles in regulating cell growth. We have introduced a multiscale model to integrate both chemical and mechanical factors and the interactions between them at the subcellular level (see Fig. 2). This chemical-mechanical model employs a subcellular element particle-based method for the mechanical submodel and a system of differential equations as the submodel for chemical signals coupled both in space and time. Details of each submodel are provided in “Methods”. In what follows, we briefly describe the coupling of submodels.

### Spatial coupling of mechanical and chemical submodels

The spatial coupling of the chemical signaling submodel and the mechanical submodel is achieved by adopting a dynamic triangular mesh over individual cells as well as the entire tissue. Such dynamic mesh is constructed using discrete nodes representing cell membranes obtained in the mechanical submodel (Fig. 2a). Shared edges and junction points between neighboring cells are identified as the edges and vertices of triangles, respectively (Fig. 6). Together with cell centers, they give rise to a triangular mesh covering individual cells (Fig. 2a, b) (More details about this mesh generator are provided in “Methods”). The chemical signaling submodel in the form of Eqs. 4–7 is then simulated over the latest mesh to reach the steady state, using an initial condition based on the old Dpp levels from the last update of the chemical signaling submodel in individual cells (Fig. 2c). Distributions of chemical signal concentrations are obtained at both individual cells and tissue level (Fig. 2d). Meanwhile, cell averages of the chemical signals are used in the mechanical submodel to direct cell growth and division.

### Temporal coupling of mechanical and chemical submodels

Cell growth and division are initiated and regulated by chemical signals. Moreover, the dynamics of chemical signals are at a much faster time scale than the time scale of mechanical changes. Therefore, quasi-steady states (Supplementary Fig. 1) of chemical signaling distributions are computed over the dynamic mesh, which captures cell and tissue deformation, and are transmitted into the mechanical submodel at some frequency. This frequency was chosen to limit redundant computation and unnecessary computational cost, as well as to transmit accurate distributions of chemical signals to the mechanical submodel. In our model, the change in the chemical signaling distribution depended on the deformation of individual cells. Therefore, to couple two submodels in time, we estimated the average time that one cell takes to enter the mitotic phase and divide. It was then converted into the frequency to update the quasi-steady state of chemical signaling distribution over the domain based on the most recent tissue configuration (More details are provided in “Methods”).

The multiscale chemical-mechanical model can be applied to study tissue development and investigate mechanisms underlying growth regulation in different biological systems. In what follows, we calibrate the model and use it to study the development of *Drosophila* wing disc pouch tissue.

### Calibration of the model for the development of *Drosophila* wing disc pouch

Dpp morphogen is the primary signal controlling cell growth and tissue development in *Drosophila* wing disc pouch^29,30,55–62^. In individual cells, the Dpp molecule binds with its receptors, Thickvein (Tkv), on the cell membrane to form the complex phosphorylates MAD (pMAD) as a downstream signal (Fig. 2c). Experimental data also suggested pMAD represses the production of Tkv as a negative feedback regulation^6^, leading to a lower synthesis of Tkv near the Dpp source region.

In the multiscale model, dynamics of the morphogen and the intracellular signaling network are modeled by a system of reaction-diffusion equations, as shown in Fig. 2c, d. Parameters ${d}_{* }$’s represent degradation rates, and v_*_’s represent production rates. v_min_ and v_max_ are the minimum and maximum production rates of Tkv receptors. The production of Dpp is modeled as a Hill function of the distance to the Dpp production region located at the AP boundary. Half of the tissue width is denoted as $L(t)$ and the width of the Dpp production region is denoted as ${r}_{s}L(t)$, where ${r}_{s}$ is a constant calibrated using experimental data^6^. The activation of intracellular signal pMAD by the binding complex Dpp-Tkv is also modeled by a Hill function, and so is the negative feedback regulation of pMAD on Tkv.

We applied a hypothesized cell division rule proposed in Wartlick et al.^30^, assuming that cells divide when the level of Dpp signal is increased by 50% compared with that at the beginning of each cell cycle, i.e.,$\,\frac{[{Dpp}]-{[{Dpp}}_{{Div}}]}{[{{Dpp}}_{{Di}v}]}\ge 50 \%$, where $[{Dpp}]$ is the Dpp concentration at the current time and ${[{Dpp}}_{{Div}}]$ is the concentration at the beginning of one cell cycle. This hypothesis, also known as the temporal model, assumes that cells have a memory to keep track of Dpp level throughout the cell cycle, and they divide once its relative change gets sufficiently large. Therefore, in our model, cells have a constant growth rate during the interphase (Supplementary Table 4), and they progress into the mitotic phase based on the division rule condition indicated above (Fig. 5a). All other parameter values were provided in Supplementary Tables 2 and 3. Movies for simulating wing disc pouch growth using this model were provided in Supplementary Information. This model can be easily revised to incorporate any other cell division rule.

### Morphogen absorbance at the tissue boundary and large decay length prolong tissue growth at a fast and spatially homogeneous rate

Dpp is generated along the midline of the wing disc and diffuses bilaterally into the neighboring tissue. Therefore, it forms an exponentially shaped gradient along the AP axis (Fig. 1b). To characterize the Dpp gradient, it is common to use a quantity called decay length ($\lambda$), which measures the distance from the source region to the location where the Dpp level is reduced to ${{\rm{e}}}^{-1}\approx 37 \%$ of its maximum (see Supplementary Information for more details)^6^. A greater decay length represents a further spread of the exponential morphogen gradient. The experimental data revealed that ubiquitous expression of Tkv led to a smaller decay length of the Dpp gradient, followed by slower growth and smaller tissue size^6^. This observation suggests that the spatial distribution of morphogen gradient should play an important role in regulating tissue growth.

**Fig. 3:**
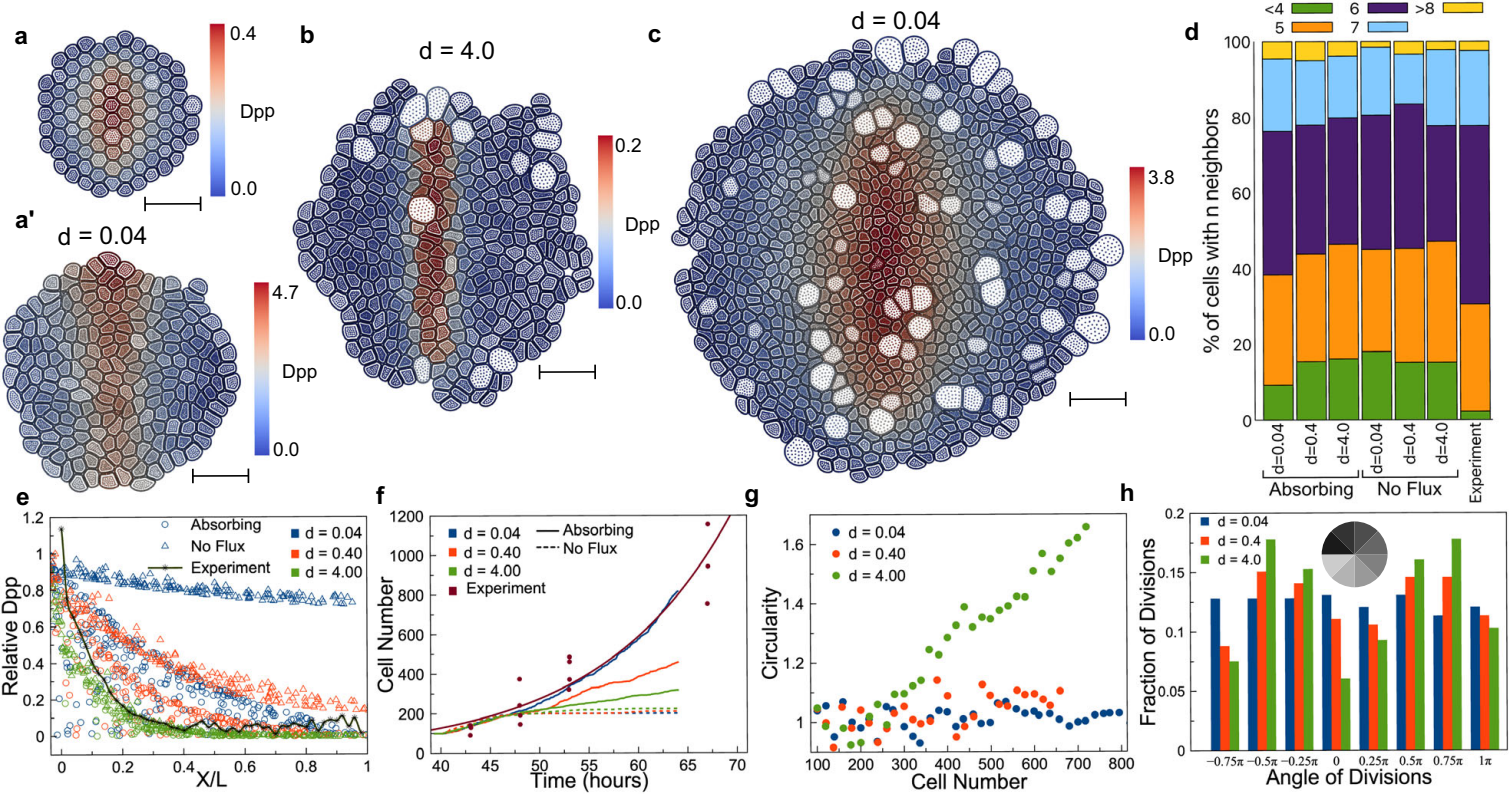
Simulation results of the coupled model with the simplified chemical submodel. **a** Initial configuration of the tissue in simulations. Final configuration of the tissue at t = 200 with **a**’ no flux boundary condition, **b** absorbing boundary condition with a large degradation rate of Dpp, and **c** absorbing boundary with a small degradation rate of Dpp. The scale bar in (**a**–**c**) is 10 μm. **d** Percentage of cells having *n* number of neighbors for simulations and experimental results. **e** Normalized Dpp profile at t = 200 with respect to the relative cell position in the tissue under different boundary conditions and with different degradation rates of Dpp. The black line shows fitted experimental quantification of the relative Dpp concentrations from 48 to 130 h. **f** Cell numbers with respect to time for different degradation rates and different boundary conditions. **g** Tissue circularity with respect to the cell number for different degradation rates. Circularity was defined as the ratio of tissue height over tissue width. **h** Distribution of the angular position of dividing cells with respect to tissue center for different degradation rates when there are 500 cells in the tissue.

To understand how the distribution of the Dpp gradient affected tissue growth, we first used a simplified chemical submodel by ignoring intracellular processes and downstream signals in the form of Eq. 3 provided in “Methods”. This simplified model allowed us to perturb the shape of the gradient easily by tuning one parameter only. In particular, the source term (second term on the right-hand side of Eq. 3) is specified as $\scriptstyle{v}_{{Dpp}}\,/\,[1+{\left(\frac{x}{{r}_{s}L\left(t\right)}\right)}^{{n}_{s}}]$ to represent the synthesis of Dpp molecules along the midline, where ${v}_{{Dpp}}$, ${r}_{s}$, ${n}_{s}$ are constants and ${\rm{L}}\left({\rm{t}}\right)$ denotes half of the tissue width. Moreover, the decay length of Dpp in this simplified model can be analytically estimated as $\scriptstyle\lambda =\sqrt{\frac{{{\rm{D}}}_{{\rm{Dpp}}}}{{{\rm{d}}}_{{\rm{Dpp}}}}}$ (See Supplementary Information for details), depending on the diffusion rate and degradation rate. A higher diffusion rate or lower degradation rate allows diffusing molecules to travel further, giving rise to a larger decay length. Both diffusion and degradation rates were calibrated to achieve a similar decay length observed in experiments^6^. We then coupled this simplified chemical submodel with the mechanical submodel under the specific cell division rule to simulate tissue growth. All simulations started with 100 cells (Fig. 3a). The final shapes of simulated tissue development are shown in Fig. 3a’, b, c. To understand how the decay length could affect tissue growth, we varied the degradation rate of Dpp concentration, which changed the underlying decay length under different boundary conditions.

First, we considered the scenario that free Dpp molecules cannot escape at the boundary of the wing disc pouch and are always kept within the tissue. This was modeled by no flux boundary condition associated with cells located at the tissue boundary. For tissue growth with no flux boundary condition, smaller degradation resulted in a flatter Dpp gradient (red and blue triangles in Fig. 3e). The Dpp concentration was saturated at high levels in individual cells, and it did not increase sufficiently to satisfy the cell division rule at the early stage of development. Therefore, most cells only experienced one cell cycle, and tissue growth stopped at the early stage, leading to small tissue sizes (red and blue dash line in Fig. 3f). With a larger degradation rate, the Dpp gradient became more exponential (green triangles in Fig. 3e), which was still far from the experimental profile of Dpp^30^ (black crosses in Fig. 3e) and the final tissue size obtained was slightly increased (green dash line in Fig. 3f). However, tissue growth was still terminated early, and the overall tissue size was much smaller than that obtained in experiments (Fig. 3f). Therefore, by assuming Dpp molecules could not escape at the boundary, tissue growth only occurred in a short time period at the early stage, and small final sizes were always obtained for different decay lengths of Dpp.

Second, we considered the scenario with Dpp being completely degraded at the periphery zone of the tissue (see “Methods” for more information), which was modeled by using absorbing boundary conditions for cells at the boundary of the tissue. Under these assumptions, the Dpp gradient changed from a linear shape to an exponential shape as the tissue size increased (indicated by circles in Fig. 3e). Furthermore, by assuming absorbing boundary conditions, the tissue growth was able to reach a size greater than the decay length of Dpp gradient (indicated by solid lines in Fig. 3f). We also observed that with a larger degradation rate, the morphogen gradient became exponential at a smaller tissue size, and the growth was maintained in a shorter period of time, giving rise to a smaller tissue size (see solid green line in Fig. 3f). With a smaller degradation rate, the morphogen gradient became exponential at a larger tissue size (blue circles in Fig. 3e) and it was still in a good agreement with experimental data^30^ (black crosses in Fig. 3e). In addition, the growth was maintained for a much longer time (Fig. 3f). To compare our simulated tissue size with experimental data more carefully, we found the time in simulations when a similar cell number was obtained as that at the initial time point and the last time point in the experiments of Wartlick et al.^30^. Then we scaled the simulation time accordingly to match those two time points and compared the cell number at intermediate time points. The simulated tissue growth matched experimental data best with the smallest degradation rate (Fig. 3f). These results were consistent with the experimental observations of a larger decay length of the Dpp, giving rise to larger tissue sizes^6^.

In addition to limited growth, it was also shown that with a higher degradation rate, the overall shape of the growing tissue, which was symmetric initially (Fig. 3a), became asymmetric, and the boundary became less smooth under the absorbing boundary condition (Fig. 3b). To look into this further, we tracked in model simulations the spatial locations of all dividing cells and visualized the distribution by dividing the tissue into eight sectors of equal size (Fig. 3h). It was observed that a higher degradation rate led to more dividing cells near the production region of Dpp, hence faster tissue growth along the AP boundary. As a result, the height of the tissue grew faster than the width, yielding an asymmetric shape (Fig. 3g). In contrast, a smaller degradation rate gave rise to more spatially homogeneous cell division (Fig. 3h) and a more symmetric overall tissue shape (Fig. 3g). Indeed, the spatially homogeneous growth rate was also observed in experiments of *Drosophila* wing disc pouch^30,63–67^, suggesting larger decay length of Dpp might be beneficial to achieve homogeneous growth in a wild-type wing disc pouch tissue. We also measured the number of neighboring cells throughout the simulated tissues with different boundary conditions and different degradation rates and compared them with experimental data (Fig. 3d). In all cases, most cells had five or six neighbors, similar to the experimental observations. With the smallest degradation rate and absorbing boundary condition, the cell population with less than four neighbors was the smallest, which was most consistent with experimental data.

Overall, simulation results suggested that the decay length of the morphogen played an essential role in maintaining tissue growth and determining the final shape when absorbing boundary condition is applied. Under such boundary conditions and the specific cell division rule, Dpp distribution with a larger decay length helped a tissue grow longer, faster, and in a more spatially homogeneous manner, which closely resembled the shape of the wild-type wing disc pouch observed in experiments. The absorbing boundary condition with a lower degradation rate allowed Dpp molecules to travel further to establish a gradient with a larger decay length, while no flux boundary condition gave rise to relatively high concentrations everywhere and much smaller tissues. In fact, the specific cell division rule was less frequently satisfied under no flux boundary conditions (see Supplementary Fig. 2 for details). In fact, it was observed that due to the hinge region around the wing disc pouch, the Dpp level dropped to almost zero at the tissue boundary^6^, suggesting the absorbing boundary condition was more biologically relevant. Therefore, the morphogen absorbance at the peripheral zone also facilitated tissue growth by reshaping the morphogen gradient in a wild-type wing disc.

### Negative feedback regulation on the synthesis of receptors promotes tissue growth through increasing morphogen decay length

It was previously shown that the transduction of Dpp signals into cells in the *Drosophila* wing disc relies on a receptor kinase Tkv. Removal of Tkv had a similar effect as the *Dpp* mutant. Recently, it was also observed that the intracellular downstream signal pMAD downregulates the production of Tkv as a negative feedback regulation, which may reshape the morphogen gradient to some extent^6^. Next, we applied our coupled model with absorbing boundary condition to study the effects of this feedback regulation on tissue growth.

Notice that in the chemical submodel, the negative feedback regulation of pMAD on Tkv was modeled using a Hill function, as illustrated in Fig. 2c. In particular, the parameter ${{\rm{k}}}_{{\rm{p}}}$ denoted the effective level of pMad involved in negative feedback regulation. Therefore, in simulations, we perturbed ${{\rm{k}}}_{{\rm{p}}}$ to give rise to different levels of this feedback regulation. Higher ${{\rm{k}}}_{{\rm{p}}}$ values gave rise to weaker negative regulation in a smaller region, while lower ${{\rm{k}}}_{{\rm{p}}}$ values led to stronger negative regulation in a larger domain. The cell division rule involved in the coupled model depended on the intracellular signal pMAD.

**Fig. 4:**
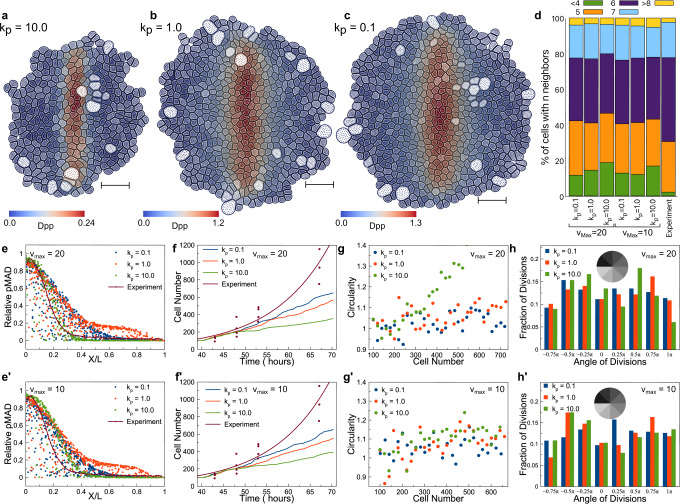
Simulation results with the advanced chemical submodel. Tissue configuration at t = 250 for ${{\rm{v}}}_{{\rm{max }}}=20$ with **a** ${{\rm{k}}}_{{\rm{p}}}=10.0$ **b** ${{\rm{k}}}_{{\rm{p}}}=1.0$ and **c** ${{\rm{k}}}_{{\rm{p}}}=0.1$. The scale bar in (**a**–**c**) is 10 μm. **d** Percentage of cells having n number of neighbors for simulations and experimental results. pMAD profile at t = 250 with respect to the relative cell position in the tissue with different levels of feedback regulation and **e** ${{\rm{v}}}_{{\rm{max }}}=20$ and **e’** ${{\rm{v}}}_{{\rm{max }}}=10$. The black line represents fitted experimental quantification of the relative signal concentrations from 48 to 130 h. Cell numbers at different levels of feedback regulation over time for **f** ${{\rm{v}}}_{{\rm{max }}}=20$ and **f’** ${{\rm{v}}}_{{\rm{max }}}=10$. Tissue circularity with respect to cell number for different levels of feedback regulation for **g** ${{\rm{v}}}_{{\rm{max }}}=20$ and **g’** ${{\rm{v}}}_{{\rm{max }}}=10$. Distributions of the angular position of dividing cells with respect to the tissue center for different levels of feedback regulation and **h** ${{\rm{v}}}_{{\rm{max }}}=20$ and **h’** ${{\rm{v}}}_{{\rm{max }}}=10$ when there are 500 cells in each simulation.

Simulations were run for low (${{\rm{k}}}_{{\rm{p}}}=10$), medium (${{\rm{k}}}_{{\rm{p}}}=1$), and high (${{\rm{k}}}_{{\rm{p}}}=0.1$) strength of negative feedback, as well as different values of maximal Tkv receptor production rate (${{\rm{v}}}_{{\rm{max }}}$) (Fig. 4). All simulations showed a good match with experimental data on the number of neighboring cells throughout tissues (Fig. 4d). The simulation results showed that, with stronger strength of negative feedback of pMAD, i.e., lower values of ${{\rm{k}}}_{{\rm{p}}}$, the tissue grew faster and the profile of cell number growing over time was closer to the one obtained in experiments (Fig. 4f, f’) and the overall tissue shape was more symmetric (Fig. 4g, g’). Moreover, the spatial distribution of dividing cells was more homogeneous (Fig. 4h, h’). However, it was also observed that simulation results for ${{\rm{k}}}_{{\rm{p}}}=0.1$ and $1$ were similar to each other. This was because the production rate of Tkv became close to the minimum almost everywhere within the tissue for sufficiently small ${{\rm{k}}}_{{\rm{p}}}$. Hence, the pMAD gradient remained more or less the same for sufficiently small ${{\rm{k}}}_{{\rm{p}}}$ values. By comparing the results generated using different values of the maximal receptor production rate (${{\rm{v}}}_{{\rm{max }}}=10$ v.s. $20$), it was observed that the effect of the feedback regulation strength became more significant when ${{\rm{v}}}_{{\rm{max }}}$ was larger, i.e., the difference in the circularity of tissue shape due to different strength of the negative feedback regulation became more visible (Fig. 4g, g’).

In fact, stronger negative feedback regulation of pMAD on Tkv receptors allowed Dpp molecules to diffuse into a larger area by reducing the binding occurrence near the Dpp production region, and therefore it gave rise to a pMAD gradient with a larger decay length (Fig. 4e, e’). Based on the cell division rule used in this study, depending on temporal changes of the Dpp signal, a more widely spreading morphogen gradient helped maintain the tissue growth for a longer time and keep the cell number increasing linearly at a faster rate, leading to a larger tissue size and more symmetric shape. This was also consistent with the results obtained by using the simplified chemical submodel (Eq. 3), in which decreasing the degradation rate led to a larger tissue size and more spatially homogeneous cell division.

## Discussion

In this paper, we described a multiscale chemical-mechanical model to study growth regulation involved in tissue development and applied it to study the development of the *Drosophila* imaginal wing disc at the larva stage. The mechanical submodel represents the shape change of individual cells and cell–cell physical interactions. It is coupled with a chemical submodel by utilizing an adaptive mesh generated over the growing tissue domain. This chemical signaling submodel describes the dynamics of the morphogen gradient and associated downstream signals inside individual cells, which control cell growth and division in the mechanical submodel. A hypothesized cell division rule based on the morphogen concentration sensed by individual cells is applied to study how the decay length of the morphogen gradient affects tissue growth.

By applying different boundary conditions in the chemical submodel, we found that tissue growth was maintained longer under absorbing boundary conditions. This indicates that the significant reduction of morphogen at the hinge region surrounding a wing disc tissue, as observed in experiments, could better promote tissue growth, compared with the case of the hinge region being a simple obstacle and preventing morphogen spread. By varying the decay length of the morphogen gradient, it was also shown that the tissue grew faster with a greater decay length. Moreover, cell division became more spatially homogeneous, giving rise to a more symmetric tissue shape consistent with experimental observations. We also found that the feedback regulation of pMAD, a downstream signal of the morphogen, on the synthesis of receptors increased the decay length and therefore facilitated tissue growth. Overall, these results suggest that the decay length of the morphogen gradient can play an important role in the growth regulation of the wing disc.

In this study, we tested a hypothesized cell division rule based on the temporal changes of morphogen, which was proposed in Wartlick et al.^30^. However, this chemical-mechanical model developed provides a general framework to study growth regulation of epithelial tissue, and it can be used to investigate other hypothesized mechanisms of growth regulation. For example, it was shown that cell mechanical stress contributes to growth control through a feedback loop in the wing disc^23,63,65^, known as the integral-feedback mechanism, which might help to achieve a more uniform growth rate in the presence of an exponential morphogen gradient. In addition, it demonstrated that cytoskeletal tension could regulate growth by altering the Hippo pathway directly^24^, working as an interaction between chemical signals and mechanical properties at a subcellular scale. Therefore, the multiscale model developed in this paper can be extended to implement cell growth rate in the form of a function of both cellular mechanical properties and chemical signals.

Moreover, it was suggested that some signaling pathways could be affected by cell mechanical properties, including shear stress and tension sensed at adherens junctions^68–70^. Meanwhile, signaling molecules could rearrange structural components within individual cells and direct new materials to the cell membrane to modify the mechanical properties^29^. These interactions between chemical and mechanical components can be incorporated into the multiscale model as more detailed experimental quantification is provided. Also, in this study, the subcellular scale was mostly used in the mechanical submodel to describe cell growth and introduce mechanical properties. Although we calculated the distributions of chemical signals over a mesh with subcellular partitioning, only cell-based averages were used to regulate cell division. However, this spatial mesh with subcellular partitioning benefited the simulation results in terms of accuracy compared with a cell-based mesh. It is also possible to apply this coupled model to study polarized chemical signals within individual cells and subcellular interaction between chemical signals and mechanical properties for other biological systems.

2D models are commonly used for studying the growth of the *Drosophila* wing disc pouch by neglecting the tissue thickness. This is because the wing disc pouch consists of epithelial cell layers, and the thickness is much smaller than the apical view dimensions. Also, the key structural components, such as E-cadherins and actomyosin, are concentrated on the top surface of the epithelia. These components contribute significantly to cell adhesion and contractility. In our 2D mechanical submodel, during cell division, the movement of the nucleus and a significant amount of cytoplasm added to the top surface of the cell, known as the process of mitotic rounding, are taken into account to include effects from the neglected dimension to some level. Additionally, a 2D model allows simulations with a large number of cells in a high resolution. As a future direction, we will try to extend our model into 3D to include extracellular matrix (ECM) and the interaction between ECM and cells, as well as intracellular signals distributed along the 3rd dimension regulating cell growth. The use of parallel computing and GPU clusters may enable 3D simulations at a similar resolution with more reasonable computational costs.

## Methods

### Mechanical submodel

**Fig. 5:**
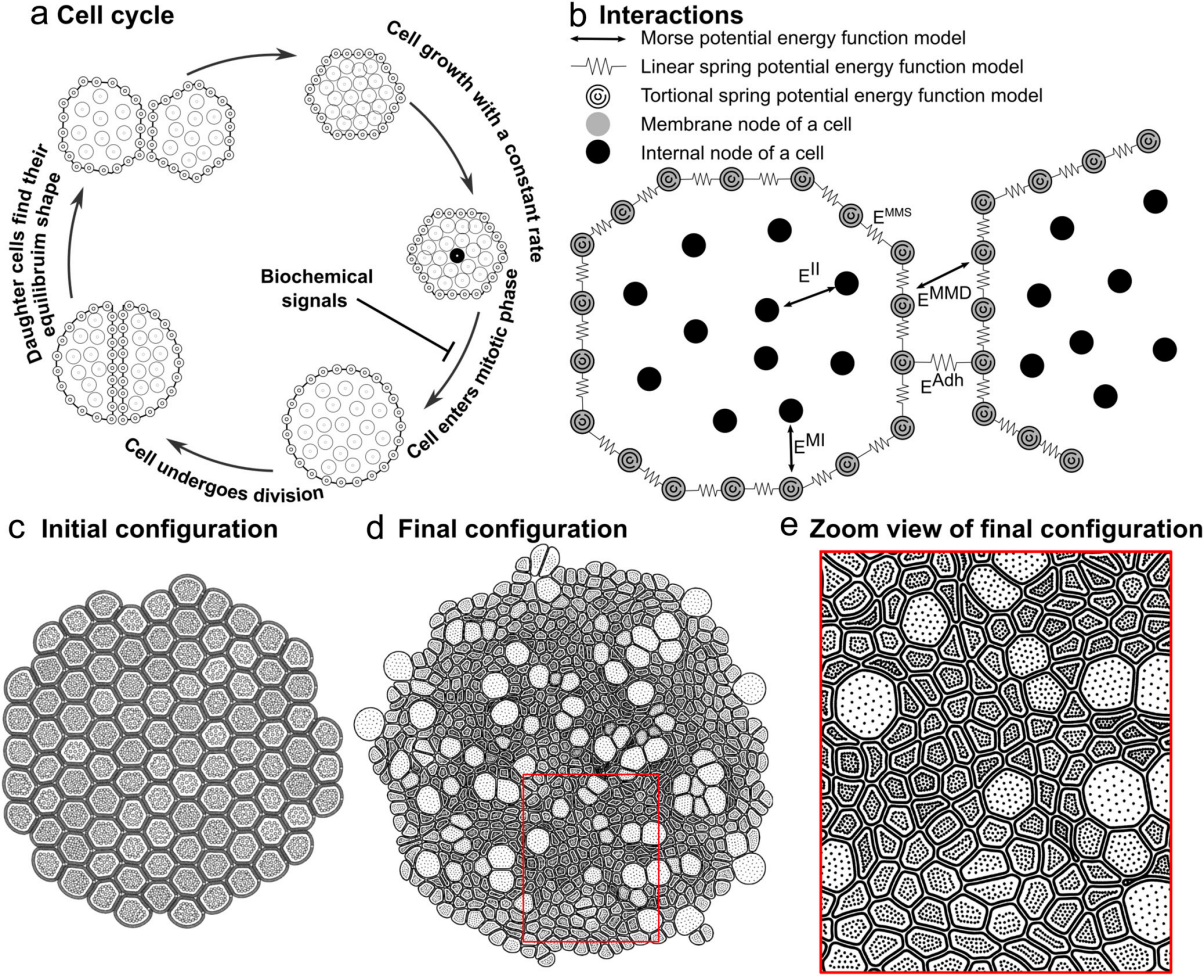
Diagram of the underlying physical basis of the mechanical submodel. **a** Life cycle of a cell in the mechanical submodel. **b** Mechanical forces between different nodes in the mechanical submodel. **c** Initial tissue configuration in a simulation with no growth regulations. **d** Final tissue configuration from the simulation in (**c**). **e** Zoom-in view of the final configuration.

For the mechanical submodel, we follow a similar approach as the Epi-scale model^46^. Epi-scale model is a multiscale subcellular element computational platform that simulates the growth of epithelial monolayers with detailed cell mechanics. Individual cells are represented as collections of two types of interacting subcellular nodes: internal nodes and membrane nodes. Internal nodes account for the cytoplasm, and the membrane nodes represent both the plasma membrane and associated contractile actomyosin cortex. Interactions between internal and membrane nodes are modeled by using different energy functions, as shown in Fig. 5b^53,71^. Combined interactions between internal nodes (${E}^{{II}}$) represent the cytoplasmic pressure of a cell. Combined interactions between internal and membrane nodes of the same cell (${E}^{{MI}}$) represent the pressure from the cytoplasm to the membrane. Interactions between membrane nodes of the same cell (${E}^{{MMS}}$) represent cortical stiffness. Cell–cell adhesion${(E}^{{Adh}}$) is modeled by combining pairwise interactions between membrane nodes of two neighboring cells. ${E}^{{MMD}}$ is a repulsive force between membrane nodes of neighboring cells and prevents membranes of different cells from overlapping. Springs and Morse energy functions are utilized to model all the interactions^54^. The following equations of the motion describe the movements of internal and membrane nodes, respectively:

1 $$\eta {\dot{x}}_{i}^{I}\,=\,-\left(\sum _{j}\nabla {E}_{{ij}}^{{MI}}+\sum _{m}\nabla {E}_{{im}}^{{II}}\right)\,i=1,2,\,\ldots ..{{N}^{I}}_{(t)}$$

2 $$\eta {\dot{x}}_{j}^{M}\,=\,-\,\left(\sum _{i}\nabla {E}_{{ij}}^{{MI}}+\sum _{k}\nabla {E}_{{kj}}^{{MMS}}+\sum _{l}\nabla {E}_{{lj}}^{{MMD}}+\nabla {E}_{j}^{{Adh}}\right)\,j=1,2,..{N}^{M}$$

where $\eta$ is the damping coefficient,$\,{x}_{i}^{I}$ and ${x}_{j}^{M}$ represent positions of internal and membrane nodes. $m$ is the index for any internal node. $k$ is the index for any membrane node of the same cell interacting with the membrane node $j$. $l$ is the index for any membrane node of a different cell interacting with the membrane node $j$. Cell growth is modeled by adding internal nodes and therefore ${N}^{I}$ increases based on the cell proliferation rate. The individual cell cycle in the current model is shown in Fig. 5a. Initial and final configurations of the tissue in a simulation with a given growth rate and cell division rate are shown in Fig. 5c–e, respectively.

### Chemical submodel

In the chemical signaling submodel, we consider a chemical signal which regulates the growth rate and cell division. A morphogen, which is a signaling molecule governing the growth and patterning of tissue development, diffuses in the extracellular space to form a gradient at the tissue level. A reaction-diffusion equation is used to model the spatiotemporal dynamics as below:

3 $$\frac{\partial \left[M\right]}{\partial t}={D}_{M}{\nabla }^{2}[M]+{s}_{M}(x)-{d}_{M}\left[M\right]$$

where $[M]$ denotes the concentration of the morphogen molecules, ${D}_{M}$ is the diffusion coefficient of morphogen molecules, and ${d}_{M}$ is the degradation rate of morphogen molecules. The production rate of morphogen molecules, varying spatially, is denoted by ${s}_{M}(x)$. ${D}_{M}$ and ${d}_{M}$ together determine how far the molecules can reach in the steady state (See Supplementary table 2–4 for more information). The local morphogen concentration is sensed by individual cells through binding with receptors on the cell membrane to activate the intracellular signaling network.

To model the intracellular signaling network, we consider the receptor ${\rm{R}}$, the complex after binding ${\rm{MR}}$, and a downstream signal ${\rm{S}}$. More components with more complex regulations can be modeled similarly. Together with the diffusive morphogen, it gives rise to a chemical signaling network at both cell and tissue levels, formulated as Eqs. 4–7 below. More specifically, the binding of the morphogen molecules and receptors is reversible, so both binding and unbinding kinetics are included with ${k}_{{on}}$ denoting the binding rate and ${k}_{{off}}$ characterizing the unbinding rate. A standard Hill function is applied to model the activation of downstream signal ${\rm{S}}$ by the complex ${\rm{MR}}$. The maximal signal production rate and concentration at which the production is half of the maximum are denoted by ${{\rm{v}}}_{{\rm{s}}}$ and ${{\rm{k}}}_{{\rm{MR}}}$, respectively. It is assumed that ${\rm{S}}$ regulates the synthesis of the receptor as a feedback regulation, which is also modeled as a Hill function, to accommodate the feedback regulation present in the *Drosophila* wing disc. The minimum and maximum receptor production rates are ${{\rm{v}}}_{{\rm{R}},{\rm{min }}}$ and ${{\rm{v}}}_{{\rm{R}},{\rm{max }}}$. The concentration producing half occupation is represented by ${{\rm{k}}}_{{\rm{s}}}$. Notice that only ${\rm{M}}$ can diffuse within the tissue, and all other components are restricted within the cell without diffusion.

4 $$\frac{\partial \left[M\right]}{\partial t}={D}_{M}{\nabla }^{2}[M]+{s}_{M}(x)-{d}_{M}\left[M\right]-{k}_{{on}}[M]\left[R\right]+{k}_{{off}}\left[{MR}\right]$$

5 $$\frac{\partial [R]}{\partial t}={v}_{R,\min }+\frac{{v}_{R,\max }-{v}_{R,\min }}{1+{\left(\frac{[S]}{{k}_{S}}\right)}^{{n}_{1}}}-{d}_{R}\left[R\right]-{k}_{{on}}[M]\left[R\right]+{k}_{{off}}\left[{MR}\right]$$

6 $$\frac{\partial [{MR}]}{\partial t}={k}_{{on}}\left[M\right]\left[R\right]-{k}_{{off}}\left[{MR}\right]-{d}_{{MR}}\left[{MR}\right]$$

7 $$\frac{\partial [S]}{\partial t}=\frac{{v}_{S}}{1+{\left(\frac{[{MR}]}{{k}_{{MR}}}\right)}^{{n}_{2}}}-{d}_{S}\left[S\right]$$

### Dynamic mesh generator to couple mechanical and chemical submodels

**Fig. 6:**
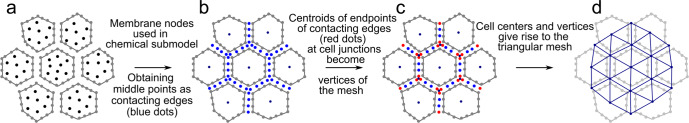
Illustration of dynamic mesh generator. **a** Nodes obtained from the mechanical submodel. Black nodes represent cytoplasm. Gray nodes represent cell membrane, connected by linear springs. **b** Identifying common edges shared by neighboring cells. Blue dots are obtained as middle points of membrane nodes from neighboring cells. **c** Identifying junction points, i.e., centroids of endpoints of contacting edges among neighboring cells, denoted by red nodes. **d** Triangles obtained by connecting cell centers and junction points.

To generate a computational mesh for the chemical signaling submodel, we first identify neighbors of individual cells based on the distance between membrane nodes of every two cells (See Fig. 6 and Supplementary Table 1 for more information). In particular, one cell is considered to be a neighbor of the other if the shortest distance between their membrane nodes is less than some threshold. This threshold is chosen based on the distance between neighboring cells obtained in the equilibrium in the simulation (See Supplementary Table 1 for more details). The same threshold is also used to determine a common edge between neighboring cells, i.e., membrane nodes from neighboring cells with a distance less than the threshold are selected to form a common edge. The middle points are calculated for each pair of those selected nodes, which give rise to a common edge between these two neighboring cells (Fig. 6b). The endpoints of each shared edge are used to determine the vertices of the triangular mesh. It is possible that multiple cells neighboring each other give rise to a junction. Therefore we consider all common edges associated with the same junction point and calculate the centroid of their endpoints near the junction as a vertex in the triangular mesh (red dots in Fig. 6c). We go over all junctions and calculate corresponding vertices throughout the tissue. Next, the center of each cell is obtained by calculating the centroid of all its membrane nodes, and it is connected to vertices obtained at junctions (Fig. 6d). By doing that, each cell is discretized by a triangular mesh that shares a common edge with its neighboring cells, and triangles in all cells give rise to a mesh covering the entire tissue (Fig. 2a). Notice that boundary cells usually lack neighbors along one or more sides; therefore their discretization will be treated separately (See the next section for more information). Nodes from cell membranes that act as the tissue boundary in those cells are selected as vertices such that some minimal distance is satisfied between successive ones. They are denoted by boundary vertices and connected with the corresponding cell centers to give rise to the triangular mesh inside boundary cells. A mesh quality check is implemented to guarantee that no highly skewed triangles are generated for convergence and accuracy of the computation over the mesh. Adjustment is conducted by merging or splitting triangles if triangles are found to be too skewed in the quality check (See the next section for more information). Such a mesh generator provides triangular meshes in individual cells, as well as a global mesh over the whole tissue. Moreover, the triangular mesh is updated at some frequency to accommodate the cell deformation and tissue growth obtained in the mechanical submodel.

### Treatments on skewed triangles and boundary cells

**Fig. 7:**
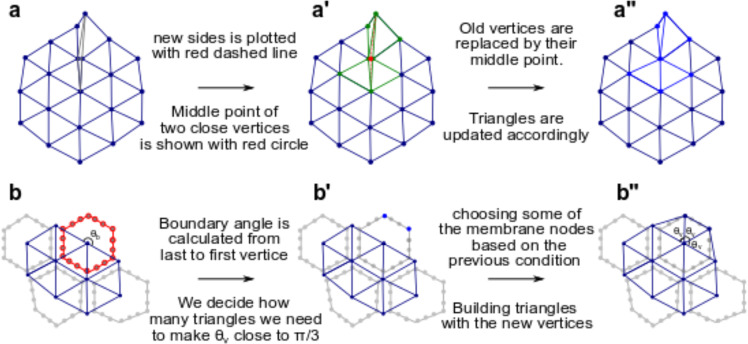
Treatments on skewed triangles and boundary cells. **a** Triangular meshes with two highly skewed triangles. **a’** Midpoint of two close vertices is calculated, and **a”** old vertices are replaced by the midpoint, then we update triangles accordingly. **b** A configuration within which the boundary cell is not covered by the triangular mesh. **b’** Some of the membrane nodes are chosen as vertices. The number of new vertices depends on the boundary angle. **b”** New triangles are built with the new vertices. Membrane nodes in (**b’**) are chosen such that these triangles are close to equilateral triangles.

Highly skewed triangles involved in the triangular mesh may affect the convergence and precision of the numerical solver. As shown in gray color in Fig. 7a, a highly skewed triangle is often generated when the shared edge of two neighboring cells is too short. To avoid that, we merge two vertices into one at the middle point when the edge of two neighboring cells is less than a threshold (${{\rm{l}}}_{{\rm{intersect}}}$) (shown in red in Fig. 7a’). Merging two vertices into one implies that each skewed triangle is now merged with the corresponding adjacent regular triangle (shown in green color in Fig. 7a’). Consequently, three connected triangles with the one skewed at the middle are now converted into two regular triangles (shown in blue color in Fig. 7a”).

Discretizing boundary cells needs special treatment since some parts of their membrane are not adjacent to any other cells. The membrane parts of boundary cells that are not adjacent to any other cells (see the cell in red color in Fig. 7b as an example) are discretized by selecting a few of their own membrane nodes (blue dots in Fig. 7b’) as the vertices of triangles. The number of membrane nodes is chosen so that triangles are close to equilateral triangles, as shown in Fig. 7b”. Mathematically, the number of new vertices on the membrane of boundary cells can be approximated according to the following equation:

8 $${n}_{{vertex}}={Round}[\,\frac{{\theta }_{{boundary}}}{\pi /3}]$$

### Discretization of governing equations of chemical submodel

We discretize Eqs. 1–4 by using the explicit Euler method in time. The diffusion of [Dpp] is approximated by passive transport between neighboring triangles. The chemical submodel is solved on the triangular mesh to obtain the quasi-steady state. The chemical signaling concentration at the cell level is obtained by calculating the average over triangles within individual cells and is also used as the initial condition for the next update on the chemical signaling distribution.

9 $$\begin{array}{l}{[{Dpp}]}_{i}^{t+\varDelta t}={[{Dpp}]}_{i}^{t}+\left(\right.{D}_{{Dpp}}\sum \limits_{{nghbr}}\frac{{A}_{i,{nghbr}}\,({\left[{Dpp}\right]}_{{nghbr}}^{t}-{\left[{Dpp}\right]}_{i}^{t})}{{{l}}_{i,{nghbr}}}\\\qquad\qquad\quad\;\;+\,\frac{{v}_{{Dpp}}}{1+{\left(\frac{{x}_{i}}{{r}_{s}L\left(t\right)}\right)}^{{n}_{s}}}\,-\frac{{d}_{{Dpp}}}{1+{\left(\frac{{x}_{i}}{{r}_{s}L\left(t\right)}\right)}^{{n}_{s}}}{\left[{Dpp}\right]}_{i}^{t} \\\qquad\qquad\quad\;\;{-\;k}_{{on}}{\left[{Dpp}\right]}_{i}^{t}{\left[{Tkv}\right]}_{i}^{t}+{k}_{{off}}{\left[{Dpp}{\_}{Tkv}\right]}_{i}^{t}\left.\right)\,\times \,\varDelta t\end{array}$$

10 $$\begin{array}{l}{\left[{Tkv}\right]}_{i}^{t+\varDelta t}={\left[{Tkv}\right]}_{i}^{t}+\left(\right.{v}_{\rm{min}}+\frac{{v}_{\rm{max}}-{v}_{\rm{min}}}{1+{\left(\frac{{[{pMad}]}_{i}^{t}}{{k}_{P}}\right)}^{{n}_{1}}}\\\qquad\qquad\quad\;\;-\,{d}_{{Tkv}}{\left[{Tkv}\right]}_{i}^{t}-{k}_{{on}}{\left[{Dpp}\right]}_{i}^{t}{\left[{Tkv}\right]}_{i}^{t}+{k}_{{off}}{\left[{Dpp}{{\_}}{Tkv}\right]}_{i}^{t}\left)\right.\,\times \,\varDelta t\end{array}$$

11 $$\begin{array}{l}{\left[{Dpp}{{\_}}{Tkv}\right]}_{i}^{t+\varDelta t}={\left[{Dpp}{{\_}}{Tkv}\right]}_{i}^{t}+\left(\right.-{d}_{{Dpp}{{\_}}{Tkv}}{\left[{Dpp}{{\_}}{Tkv}\right]}_{i}^{t}\\\qquad\qquad\qquad\quad+\,{k}_{{on}}{\left[{Dpp}\right]}_{i}^{t}{\left[{Tkv}\right]}_{i}^{t}-{k}_{{off}}{\left[{Dpp}{{\_}}{Tkv}\right]}_{i}^{t}\left)\right.\,\times \,\varDelta t\end{array}$$

12 $${[{pMad}]}_{i}^{t+\varDelta t}={[{pMad}]}_{i}^{t}+\left(\frac{{v}_{P}}{1+{\left(\frac{{[{Dpp}{{\_}}{Tkv}]}_{i}^{t}}{{k}_{{Dpp}{{\_}}{Tkv}}}\right)}^{{n}_{2}}}-{d}_{P}{\left[{pMad}\right]}_{i}^{t}\right)\,\times \,\varDelta t$$

In Eqs. 9–12, ${[* ]}_{{\rm{i}}}^{{\rm{t}}}$ is the concentration of chemical signals on triangle ${\rm{i}}$ at time ${\rm{t}}$. The diffusion is approximated as the flux between two neighboring triangles, which is dependent on the length of the contact edge, ${{\rm{A}}}_{{\rm{i}},{\rm{nghbr}}}$, and the concentration gradient between them. The steady state is obtained when the relative difference in concentrations of each chemical signal (Dpp, Tkv, and pMad) between two successive time steps is less than ${10}^{-4}$, i.e.,

13 $$\frac{{[\alpha ]}_{i}^{t+1}-{[\alpha ]}_{i}^{t}}{{[\alpha \,]}_{i}^{t}\,\Delta t} < {10}^{-4}\,\forall i\in {all}\,{meshes},\,\alpha =\,{Dpp},\,{Tkv},\,{Dp}{p}_{{Tkv}},\,{pMad}$$

Absorbing boundary condition is applied by assuming that free Dpp molecules would be diminished to zero at the boundary of the domain, corresponding to the fact that no Dpp signal was captured in the hinged region surrounding the wing disc pouch. We impose this condition in our chemical submodel by enforcing zero Dpp level on triangles along the tissue boundary at every time step, i.e.,

14 $${[Dpp]}_{i}^{t}=0\forall t\,\& \,\forall i\in boundary\,meshes$$

### Frequency of information exchange between mechanical and chemical submodels

When coupling the mechanical and chemical submodels, cell configurations used in the chemical submodel and chemical signal concentration used in the mechanical submodel need to be updated at some frequency to ensure consistent information is used in both submodels. Such frequency has to be chosen appropriately because too small frequencies will lead to non-compatible information exchanged between two submodels, while too big frequencies result in redundant computation and high computational cost.

When applying the coupled model to study the development of the *Drosophila* wing disc, the minimum time scale that takes one cell to enter the mitotic phase and divide is used to estimate the frequency of information exchange between two submodels. Considering the maximum cell growth rate at the beginning of the simulation (g_0,max_ = 1.1 × 10^−4^), it takes at least 9090 units of time in the mechanical submodel for one cell to start a cell cycle and divide. Note that the growth rate of daughter cells decays with respect to time. Therefore, cell cycle length increases in the later stage of the simulation. Thus 9090 units of time is the shortest cell cycle used in the simulation. Also, the chance of getting cell divisions will be higher if there are more cells involved. Therefore, we update the steady state of chemical signal concentration based on the cell configurations obtained most recently in the mechanical submodel every 200 units of time in the mechanical submodel, i.e., the coupling frequency ${{\rm{f}}}_{{\rm{exch}}}=0.005$. This means we update the profile of chemical signals around 45 times within each cell cycle. This estimated frequency allows us to compute the relative change on the Dpp signal (Eq. 5) for all cells without too expensive computational cost.

We have utilized Intel(R) Xeon(R) CPU E5-1650 v2 3.50 GHz CPU and an Nvidia TITAN V graphic card (GPU) to run our simulations. The GPU is used to simulate the mechanical submodel, whereas the CPU is utilized to calculate the pseudo-steady state of the morphogen profile in the chemical signaling submodel. Typically, a simulation of the simplified model takes around 100 h to complete, while a simulation of the advanced model takes approximately 200 h. These simulation times are obtained from simulations with the highest rate of cell division in both simplified and advanced models. Other simulations in each model take less time to complete as they have fewer cells included in the model during the simulation.

### Reporting summary

Further information on research design is available in the Nature Research Reporting Summary linked to this article.

## Supplementary information

Supplementary Materials

Reporting Summary

Supplementary Information Movie 1

Supplementary Information Movie 2

Supplementary Information Movie 3

Supplementary Information Movie 4

Supplementary Information Movie 5

Supplementary Information Movie 6

Supplementary Information Movie 7

Supplementary Information Movie 8

## Data Availability

Data sets generated by this work can be accessed at https://github.com/weitaoc/EpiScale_Signal.git or in Zenodo (10.5281/zenodo.7947744).

## Code availability

Simulation codes written in C++ can be accessed at https://github.com/weitaoc/EpiScale_Signal.git or in Zenodo (10.5281/zenodo.7947744).
