# Supplementary material for: A multiscale chemical-mechanical model predicts impact of morphogen spreading on tissue growth
Source: NPJ Syst Biol Appl. 2023 May 20;9:16. doi: 10.1038/s41540-023-00278-5 (PMC10199952; doi:10.1038/s41540-023-00278-5)
Supplement: Supplementary file 1 — Supplementary Materials [file 41540_2023_278_MOESM1_ESM.pdf]

Supplementary Information

A Multi-scale Chemical-Mechanical Model Predicts Impact of Morphogen Spreading on Tissue Growth

Alireza Ramezani[1,3], Samuel Britton[2], Roya Zandi[1,3], Mark Alber[2,3], Ali Nematbakhsh*[2], Weitao Chen*[2,3]

1 Department of Physics and Astronomy, University of California, Riverside, CA 92521, USA

2 Department of Mathematics, University of California, Riverside, CA 92521, USA

3 Interdisciplinary Center for Quantitative Modeling in Biology, University of California, Riverside, CA 92521, USA

* Corresponding Authors: nematba@ucr.edu, weitaoc@ucr.edu

Contents

S1 Convergence tests on the chemical signaling submodel. 2

S2 Parameters used in mesh generation method 4

S3 Analytical solution to the 1D diffusion equation 4

S4 Comparison of Dpp Relative Change under Different Boundary Conditions 6

S5 Table of parameters 6

S6 References 8

S1 Convergence tests on the chemical signaling submodel.

To verify the convergence of the numerical method to approximate the quasi-steady state in the chemical submodel, we solved the reaction-diffusion equation describing the morphogen dynamics within a fixed domain at different mesh sizes. Since we assumed the apical view of wing disc tissue was symmetric, for simplicity, only the right-hand side of the tissue domain was considered in this test. Without loss of generality, we first tested convergence on mesh size using the simplified model (Eqn. 13). The absorbing boundary condition was applied at the right end, and the Euler method was applied as the time integration method. Therefore, the equation we solved was

$$\frac{\partial M}{\partial t} = D\nabla^2 M + s(x) - dM, \qquad \text{(Eqn. S1)}$$

where M denoted the concentration of the morphogen molecules, D was the diffusion coefficient of morphogen molecules, d was the degradation rate of morphogen molecules. The production rate of morphogen molecules by cells, denoted by $s(x)$, varied spatially. In particular, it was zero almost everywhere except at the origin (Supplementary Figure 1A and Supplementary Figure 1B). The 1D domain was divided into N=10 and 20 equal subintervals and the steady state of Eqn. S1 was obtained on those two meshes. It was observed that the decay length of the morphogen gradient, $\lambda = \sqrt{\frac{D}{d}}$ (see Section S5 for more detail), which measured the spread of the morphogen, was independent of the spatial mesh size (Supplementary Figure 1A' and Supplementary Figure 1B'), indicating the convergence of the morphogen gradient when reducing spatial mesh size.

We also compared the relative change of Dpp on meshes with different sizes in a 2D domain. Since the cell division rule we employed in the coupled model was dependent on the relative change, it was necessary to show that the relative change was independent of mesh size, and consequently the cell division was independent of the mesh size. In particular, we chose a tissue configuration with 200 cells obtained from the simulation of the coupled model and applied the mesh generator to it to obtain a triangular mesh. We calculated the quasi-steady state of the morphogen gradient following Supplementary Eqn. 1. After that, the computation was repeated over the tissue with 210 cells generated by the same simulation of the coupled model. Then the relative change in the Dpp level between those two stages was calculated following Eqn. 5. We then split each triangle in the meshes for 200 and 210 cells into 2 or 4

smaller triangles by dividing edges along the cell boundary into 2 or 4 equal segments. Relative changes on Dpp were obtained following the same computation over those two refined meshes. The results are shown in Supplementary Figure 1C. The relative changes obtained over different meshes had very similar distributions. In particular, if comparing the mesh without splitting with the one having mesh size halved, the R-squared was 0.9 with a slope of 0.89 and an intercept of 0.005 for the two data sets of relative changes. If comparing those two refined meshes, the R-squared was 0.98 with a slope of 1.2 and an intercept of 0.009. Both comparisons showed the relative changes of Dpp obtained over different meshes were similar to each other, indicating the approach we used to calculate such relative change was robust to the mesh size.

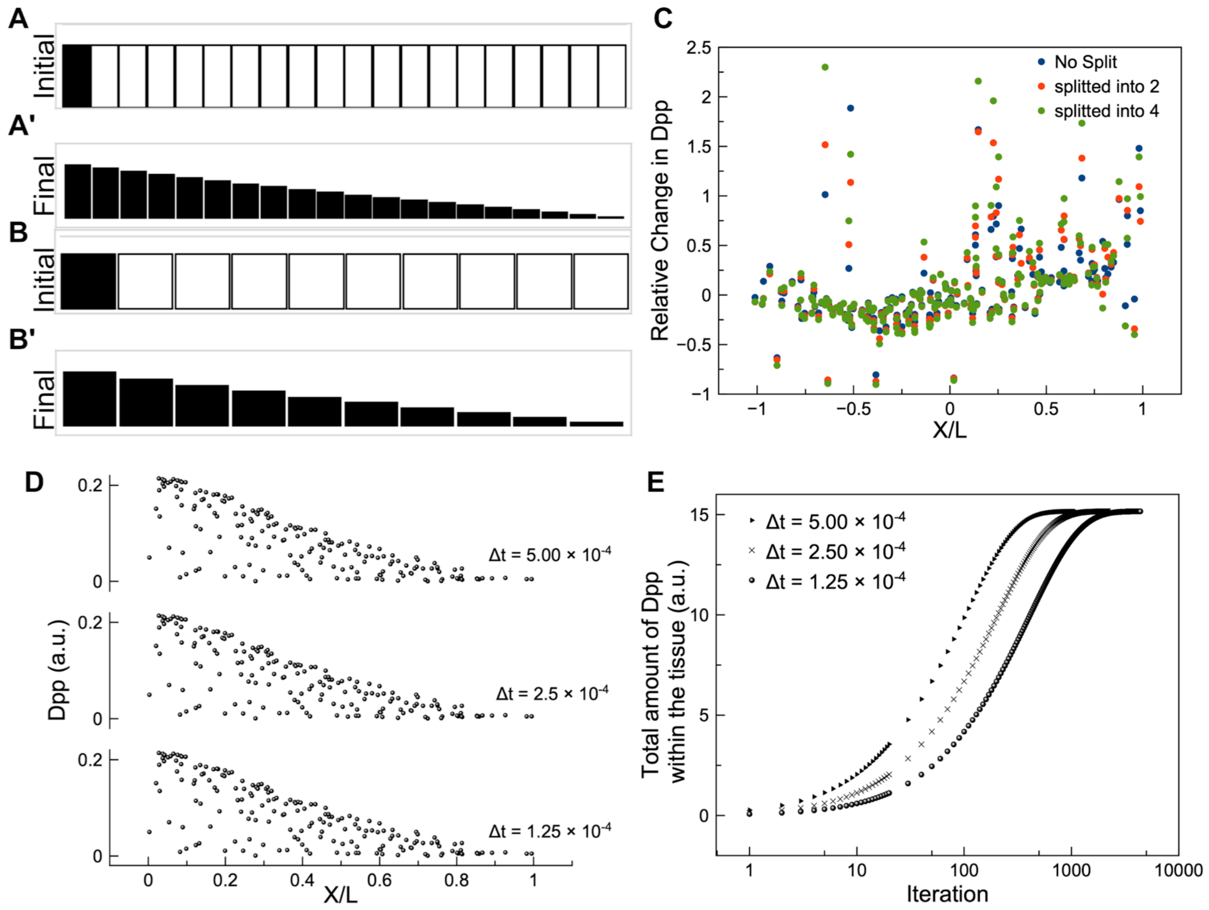

Supplementary figure 1. Initial distribution of morphogen in 1D domain using A) 20 and B) 10 mesh grids. A') and B') show the final distributions for (A) and (B) accordingly. Refer to Supplementary table 2 for parameters (d=0.04). C) Relative change in Dpp profile for different mesh sizes. D) Cell level Dpp profile calculated based on the simplified model using different time steps and E) Tissue level Dpp calculated as a function of the number of iterations for different time steps.

We also verified the convergence of the morphogen gradient with respect to time step size in the 2D domain by choosing d=0.04, the final time to be 50 and a total of 182 cells. The quasi-steady-state level of Dpp was calculated using the time step $\Delta t = 5\times10^{-4}$. We reduced the time step by a factor of 2 and 4 and confirmed that very similar morphogen gradients were achieved

(Supplementary Figure 1D). However, more iterations were required to reach the quasi-steady state (Supplementary Figure 1E), indicating the convergence when reducing the time step size.

S2 Parameters used in mesh generation method

Notice that in the mechanical submodel, the distance between membrane nodes of neighboring cells is about $0.3\ \mu m$ on average. Therefore, the threshold for determining whether two cells are neighbors, denoted by l_{thres}, should be greater than $0.3\ \mu m$. On the other hand, l_{thres} should be small enough to distinguish membrane nodes from neighboring cells. To satisfy both constraints, we choose l_{thres} to be $0.5\ \mu m$. The same threshold is used to merge vertices that are close to each other into one, as discussed in Methods, and it is denoted by $l_{intersect}$. We allow at least $1.0\ \mu m$ distance between two neighboring vertices to avoid skewed triangles in the mesh.

Within the tissue simulated, it is commonly observed that three cells are neighbors to each other, and there are also a few cases with four cells being neighbors to each other. Different threshold values must be used to determine vertices shared by three or four cells in the mesh structure. More specially, $l_{thres} = 0.5\ \mu m$ is used for three-cell cases, and $l_{thres-4\ cell} = 0.35\ \mu m$ is applied for four-cell cases since a higher resolution is required to identify neighboring cells. A list of parameters used for discretization is shown as below:

Parameter	Value	Units	Source or calibration section
$\boldsymbol{l_{thres}}$	0.5	μm	Based on the resolution of the model
$\boldsymbol{l_{intersect}}$	0.5	μm	Based on the resolution of the model
$\boldsymbol{l_{thres-4\ cell}}$	0.35	μm	Based on the resolution of the model

Supplementary table 1 – Parameters used in discretization in the mechanical and chemical submodels

S3 Analytical solution to the 1D diffusion equation

The simplified chemical submodel, in which only one chemical signal is considered, can be solved analytically on the 1D domain. Such analytical solutions can help to gain insights into how different parameters affect the steady state distribution.

The reaction-diffusion equation with a specific source function can be written as below:

$$\frac{\partial M}{\partial t} = D\nabla^2 M - dM + s(x) \qquad \text{(Eqn. S2)}$$

$$s(x) = \begin{cases} v & x < L_s \\ 0 & x > L_s \end{cases} \qquad \text{(Eqn. S3)}$$

where M is the morphogen concentration, D is the diffusion rate and d is the degradation rate. Production function, denoted by $s(x)$, is nonzero only near the origin. L_s is the length of the source region

For steady state solution satisfying $\frac{\partial M}{\partial t} = 0$, we can assume it in the form of

$$M(x) = c_1 e^{\frac{-x}{\lambda}} + c_2 e^{\frac{x}{\lambda}} \qquad \text{(Eqn. S4)}$$

where $\lambda = \sqrt{\frac{D}{d}}$. Boundary conditions are applied to determine values for c_1 and c_2. Assuming M is zero at infinity, the following boundary condition can be used and c_2 can be determined.

$$M(\infty) = 0\,; c_2 = 0 \qquad \text{(Eqn. S5)}$$

On the other hand, the total amount of M within the domain should be equal to the net change due to the production and degradation based on the mass conservation, i.e.,

$$\int_0^\infty M(x)\,dx = c_1\sqrt{\frac{D}{d}} = \frac{L_s v}{d} \qquad \text{(Eqn. S6)}$$

Therefore, c_1 can be determined, and the analytical solution is written as below:

$$M(x) = \frac{L_s v}{\sqrt{Dd}} e^{-\sqrt{\frac{d}{D}}x} \qquad \text{(Eqn. S7)}$$

From this equation, we observe that the spread of M depends on diffusion rate and degradation rate, i.e. $\sqrt{\frac{D}{d}}$, which is defined as the decay length. In particular, the concentration of M spreads further if the decay length is larger.

When considering a finite domain with length L, the following boundary condition is used and $c_2$can be determined.

$$M_{(x=L)} = 0;\ c_2 = -c_1 e^{\frac{-2L}{\lambda}} \qquad \text{(Eqn. S8)}$$

Similar to the previous calculation, we can determine c_1 by integrating both sides of the reaction-diffusion equation,

$$\int_0^L (D\nabla^2 M - dM + s(x)\,)dx = 0 \qquad \text{(Eqn. S9)}$$

Therefore c_1 and the steady state solution are

$$c_1 = \frac{L_s v}{d\lambda(e^{\frac{-L}{\lambda}} - 1)^2} \qquad \text{(Eqn. S10)}$$

$$M(x) = \frac{L_s v}{d\lambda(e^{\frac{-L}{\lambda}} - 1)^2}\left(e^{\frac{-x}{\lambda}} - \frac{e^{\frac{x}{\lambda}}}{e^{\frac{2L}{\lambda}}}\right) \qquad \text{(Eqn. S11)}$$

Based on this result, we observe that the steady state profile is closer to an exponential function when $\lambda << L$. When $\lambda >> L$, it decays linearly.

S4 Comparison of Dpp Relative Change under Different Boundary Conditions

No flux boundary condition gives rise to a flatter Dpp gradient when the degradation rate is very small. However, the level of Dpp gradient becomes high everywhere. As shown in Figure 5A, in the coupled model, the cell division condition will be checked multiple times in a cell cycle, and cells will divide only if the relative increase of Dpp is greater than 50%. So reaching a high level of Dpp doesn't guarantee multiple times of cell division. To compare cell divisions under those two boundary conditions more carefully, we computed the relative change of Dpp in individual cells for simulated tissues with 200-220 cells under different conditions, and plotted the relative change with respect to their relative cell positions along the x-axis, as shown in Supplementary Figure 3. Compared with absorbing boundary condition, under no flux boundary condition the relative increase of Dpp level is lower than 50% in most cells, except some near the middle of the tissue. This is because the absolute Dpp level is high everywhere and the relative increase becomes small, preventing cell division under no flux boundary condition. Therefore, Dpp distribution with a larger decay length helps a tissue grow longer, faster, and in a more spatially homogeneous manner, is only true for absorbing boundary conditions where the Dpp concentration is always decaying from the source to the sink.

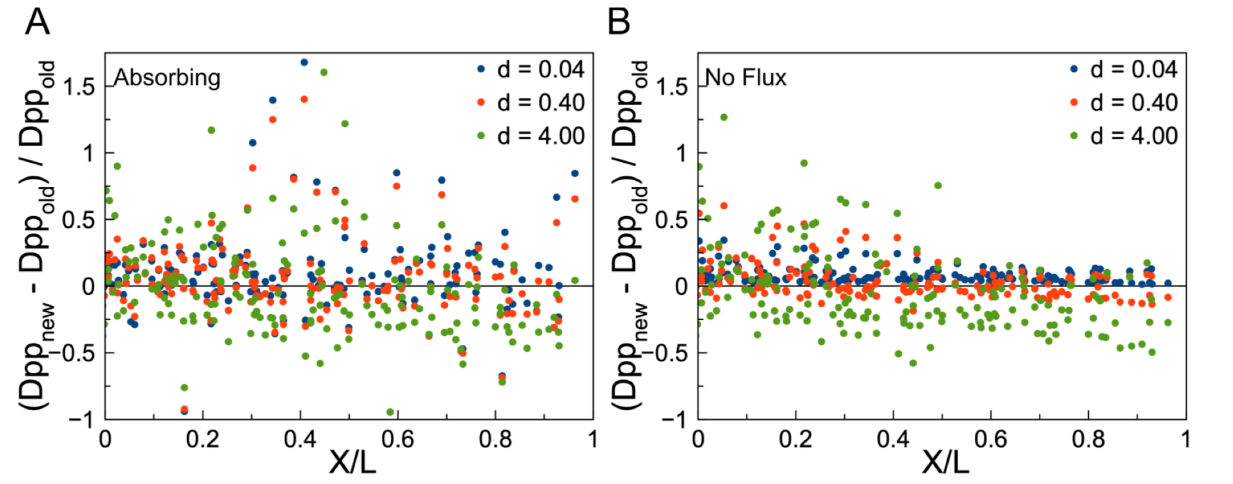

Supplementary figure 2. Relative change of Dpp value at cell level. The relative change in Dpp value calculated as the tissue grew from 200 to 220 cells under two boundary conditions: A) absorbing and B) no flux. The values are presented as a function of relative distance from the source region to individual cells. Tissue configurations are taken from the simulation result of a simplified model using absorbing boundary condition and d=0.04. Different degradation rates are tested under different boundary conditions.

S5 Table of parameters

Parameters related to the mechanical submodels, mechanical submodel, and coupling are listed below.

Parameter	Value	Units	Source or calibration section
$\boldsymbol{D_{Dpp}}$	20.0	$\mu m^2.s^{-1}$	(Zhu et al., 2020)
$\boldsymbol{v_{Dpp}}$	1.0	$M.s^{-1}$	(Zhu et al., 2020)

d_{Dpp}	0.04 & 0.4 & 4.0	s^{-1}	Parameter study
r_s	0.12	_	(Zhu et al., 2020)
Δt	5×10^{-4}	s	Based on stability of algorithm
f_{exch}	0.005	s^{-1}	Based on cell cycle lifetime

Supplementary table 2 – Parameters in simplified chemical submodel in Eqn 8. We studied the effect of degradation of Dpp on tissue growth, proliferation distribution of cells within the tissue, and tissue circularity.

Parameter	Value	Units	Source or calibration section
d_{Dpp}	0.1	$\mu m^2.s^{-1}$	Calibration in this study
k_{0n}	0.025	$M^{-1}.s^{-1}$	(Zhu et al., 2020)
k_{0ff}	0.000025	$M^{-1}.s^{-1}$	(Zhu et al., 2020)
n_s	40.0	_	(Zhu et al., 2020)
v_{min}	1.0	$M.s^{-1}$	(Zhu et al., 2020)
v_{max}	10 & 20	$M.s^{-1}$	Parameter study
d_{Tkv}	0.1	s^{-1}	(Zhu et al., 2020)
k_p	0.1 & 1.0 & 10	M	Parameter study
n_1	8.0	_	(Zhu et al., 2020)
d_{Dpp_Tkv}	0.1	s^{-1}	(Zhu et al., 2020)
v_{pMad}	1.0	$M.s^{-1}$	(Zhu et al., 2020)
d_{pMad}	0.1	s^{-1}	(Zhu et al., 2020)
k_{Dpp_Tkv}	1.0	M	(Zhu et al., 2020)
n_2	−2.0	_	(Zhu et al., 2020)

Supplementary table 3 – Parameters in the advanced chemical submodel. Parameters not included are the same as the simplified model. kp and v_{max} are perturbed to study the effect of feedback strength on tissue growth.

The mechanical submodel has been calibrated in Nematbakhsh et al., (2017). We used the same mechanical parameters that can describe the mechanical properties of cells in *Drosophila* wing disc. The growth related parameters have been reduced in this study to reach better convergence, as shown in Supplementary table 4. The proliferation rate of the cell i^{th} is chosen stochastically between the minimum ($g_{0,min}$) and maximum ($g_{0,max}$) growth speed to resemble randomness in the cell growth rate. The maximum and minimum growth speed decay exponentially with respect to time to resemble growth speed reduction in the wing disc in time.

$$g_i(t) = Rnd[g_{0,min}, g_{0,max}]e^{-k_g t} \quad \text{(Eqn. S12)}$$

$$g_i(t) = (g_{0,Avg} + Rnd[-g_0, g_0])e^{-k_g t} \quad \text{(Eqn. S13)}$$

Parameter	Value	Units*	Source or calibration section
$\boldsymbol{g_{0,min}}$	0.55×10^{-4}	$(a.u)^{-1}$	Model convergence
$\boldsymbol{g_{0,max}}$	1.1×10^{-4}	$(a.u)^{-1}$	Model convergence
$\boldsymbol{k_g}$	1.1×10^{-5}	$(a.u)^{-1}$	–

Supplementary table 4 – Parameters in the mechanical submodel. * Arbitrary unit of time.

S6 References

Nematbakhsh, A., Sun, W., Brodskiy, P. A., Amiri, A., Narciso, C., Xu, Z., Zartman, J. J. and Alber, M. (2017). Multi-scale computational study of the mechanical regulation of cell mitotic rounding in epithelia. *PLOS Comput. Biol.* **13**, e1005533.

Zhu, Y., Qiu, Y., Chen, W., Nie, Q. and Lander, A. D. (2020). Scaling a Dpp Morphogen Gradient through Feedback Control of Receptors and Co-receptors. *Dev. Cell* **53**, 724-739.e14.
